# Myocardial inefficiency is an early indicator of exercise-induced myocardial fatigue

**DOI:** 10.3389/fcvm.2022.1081664

**Published:** 2023-01-11

**Authors:** Christine Bjørkvik Erevik, Øyunn Kleiven, Vidar Frøysa, Magnus Bjørkavoll-Bergseth, Monica Chivulescu, Lars Gunnar Klæboe, Lars Dejgaard, Bjørn Auestad, Øyvind Skadberg, Tor Melberg, Stig Urheim, Kristina Haugaa, Thor Edvardsen, Stein Ørn

**Affiliations:** ^1^Department of Cardiology, Stavanger University Hospital, Stavanger, Norway; ^2^ProCardio Center for Innovation, Department of Cardiology, Oslo University Hospital, Rikshospitalet, Oslo, Norway; ^3^Department of Mathematics and Physics, University of Stavanger, Stavanger, Norway; ^4^Research Department, Stavanger University Hospital, Stavanger, Norway; ^5^Department of Biochemistry, Stavanger University Hospital, Stavanger, Norway; ^6^Department of Cardiology, Bergen University Hospital, Bergen, Norway

**Keywords:** myocardial work, sports cardiology, exercise, left ventricular function, myocardial strain, exercise-induced cardiac fatigue, myocardial efficiency, athletes heart

## Abstract

**Background:**

The effect of prolonged, high-intensity endurance exercise on myocardial function is unclear. This study aimed to determine the left ventricular (LV) response to increased exercise duration and intensity using novel echocardiographic tools to assess myocardial work and fatigue.

**Materials and methods:**

LV function was assessed by echocardiography before, immediately, and 24 h after a cardiopulmonary exercise test (CPET) and a 91-km mountain bike leisure race. Cardiac Troponin I (cTnI) was used to assess myocyte stress.

**Results:**

59 healthy recreational athletes, 52 (43–59) years of age, 73% males, were included. The race was longer and of higher intensity generating higher cTnI levels compared with the CPET (*p* < 0.0001): Race/CPET: exercise duration: 230 (210, 245)/43 (40, 45) minutes, mean heart rate: 154 ± 10/132 ± 12 bpm, max cTnI: 77 (37, 128)/12 (7, 23) ng/L. Stroke volume and cardiac output were higher after the race than CPET (*p* < 0.005). The two exercises did not differ in post-exercise changes in LV ejection fraction (LVEF) or global longitudinal strain (GLS). There was an increase in global wasted work (*p* = 0.001) following the race and a persistent reduction in global constructive work 24 h after exercise (*p* = 0.003).

**Conclusion:**

Increased exercise intensity and duration were associated with increased myocardial wasted work post-exercise, without alterations in LVEF and GLS from baseline values. These findings suggest that markers of myocardial inefficiency may precede reduction in global LV function as markers of myocardial fatigue.

## Introduction

Increased physical activity increases health benefits (1–4). However, prolonged high-intensity endurance exercise may be associated with an increased risk of adverse clinical outcomes (5) and alterations in left and right ventricular (LV and RV) function (6, 7). These exercise-induced alterations in myocardial function are often called exercise-induced cardiac fatigue (8–10).

Numerous studies have reported on post-exercise LV function assessed by echocardiography (8). A large meta-analysis reported an overall reduction in LV global longitudinal strain (GLS), LV ejection fraction (EF), and diastolic function after bouts of prolonged, high-intensity exercise exceeding 2 h of duration (8). However, the studies included in the meta-analysis did not compare the response to different exercise intensities and durations within the same individuals. Furthermore, echocardiographic parameters, such as LV volumes and GLS, are influenced by pre- and afterload, potentially precluding the assessment of the myocardial response to exercise (11, 12).

A novel echocardiographic method has been developed to assess non-invasive parameters of myocardial work (MW) (13–16). MW uses a combination of LV GLS and systolic blood pressure (SBP) to obtain LV pressure-strain loops. These MW parameters offer new insights into alterations in LV function following exercise compared with traditional echocardiographic parameters. Moreover, since MW parameters can identify pathological alterations in myocardial function due to ischemia, these parameters may present new insights into exercise-induced cardiac fatigue (17, 18).

This study aimed to determine the LV response to increased exercise workload by comparing two exercises of different exercise intensity and duration, using both traditional and MW echocardiographic parameters before and after exercise.

## Materials and methods

Study participants were recruited among healthy, prior participants in the NEEDED 2013 and 2014 studies (19, 20). Exclusion criteria were age below 18 years of age, any prior history, signs or symptoms of cardiac disease, pathological ECG or echocardiographic findings, and a normal CT coronary angiography, without obstructive CAD following exercise.

**FIGURE 1:**
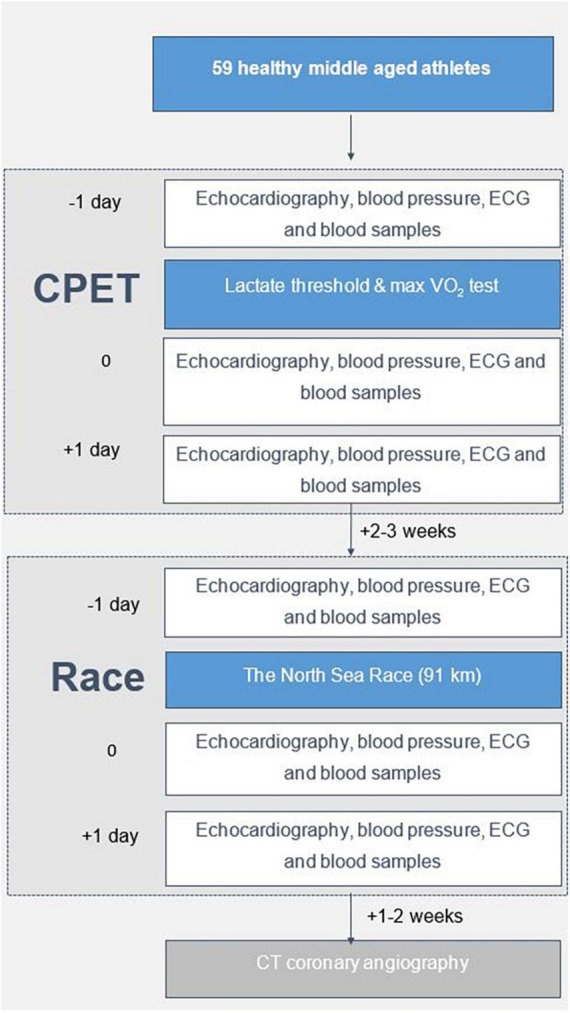
Flow chart of the study.

The present study was conducted in 2018. Echocardiography was acquired before exercise, immediately after, and at 24 h following two episodes of high-intensity endurance exercise in 2018 (Figure 1). The first exercise was a cardiopulmonary exercise test (CPET) (Supplementary material 1). The second exercise was participating in the 91-km North Sea Race Mountain bike leisure race in 2018. Cardiac Troponin I (cTnI) was used as a biomarker of myocardial stress (21). Blood samples were acquired before (baseline) and at 3-h (the expected maximum elevation) and 24 h (to assess recovery) following both exercises. During the race, power meters (Stages Power Meters, Boulder, CO, US) were used to assess the amount of work performed. Exercise intensity was assessed by continuous heart rate measurement using Garmin heart rate straps and Garmin Forerunner 935 Sport Watch (Garmin, Olathe, KS, USA). A CT coronary angiography was performed 2–3 weeks after the race in 2018 to ensure the absence of new coronary artery obstruction potentially influencing the echocardiographic assessments and cTnI response. Informed consent was obtained from all study participants prior to study inclusion. The Regional Ethics Committee approved the study (REK nr 2013/550).

### Echocardiographic image acquisition

GE Vivid E 95 ultrasound systems and 4V probes (Vingmed Horten, Norway) were used for all echocardiographic assessments. Three medical doctors were responsible for acquiring images in relation to the CPET, and six medical doctors worked on parallel stations in relation to the cycle race to acquire images immediately after finishing the race. Comprehensive imaging protocols were applied. Appropriate frame rates were applied, to allow later high-quality post-processing, including speckle-tracking strain analysis.

### Analysis of myocardial function and morphology

All echocardiographic analyses were performed offline on EchoPAC V202 (General Electric Vingmed Ultrasound AS) by a researcher blinded to clinical data and exercise information. All echocardiographic parameters were calculated according to the European Association of Cardiovascular Imaging (22, 23). The Devereux formula was used to determine LV mass. Mitral valve inflow was assessed using pulsed-wave Doppler at the mitral valve leaflets; pulsed-wave tissue Doppler imaging was performed to assess septal mitral annular velocity. LV volumes and EF were assessed using three-dimensional imaging. Two-dimensional speckle tracking imaging was used to study LV deformation. LV GLS was calculated as an average longitudinal strain value based on apical two-, three-, and four-chamber views at a 60–70 frames/second frame rate using the automated function imaging (AFI). Time to peak strain was defined as the time from onset Q/R wave on ECG to peak negative longitudinal strain during the entire cardiac cycle. Post-systolic strain was calculated as the absolute difference between peak global longitudinal strain (peak G) and peak longitudinal strain during systole (peak S). Post-systolic index (PSI) was defined as [(peak G–peak S)/peak G] × 100. Early systolic lengthening was determined in 18 left ventricular segments, and peak P represents the maximal positive strain value in early systole. Mechanical dispersion (MD) was defined as the standard deviation of time to peak negative strain in 18 left ventricular segments.

### Myocardial work (MW) analysis

Myocardial work (MW) calculation was performed offline using EchoPAC software version 202 (General Electric Vingmed Ultrasound AS). A commercially available algorithm was used to calculate four MW parameters (see below). The LV pressure curve was estimated using an empiric reference pressure curve that was adjusted according to the duration of the isovolumic and ejection phases. SBP was measured with a brachial cuff with study subjects sitting. The opening and closure of the aortic and mitral valves were determined manually in the three- and four-chamber apical views based on Doppler signals and visualization of valve opening and closure.

Pressure strain was generated in each myocardial segment by the EchoPAC software, and global values were calculated as mean values of all segments. The global work index (GWI) parameter is the total work performed by the left ventricle, using the area of the pressure-strain loop between the mitral valve closure and opening. Global constructive work (GCW) is the sum of positive work due to myocardial shortening in systole and negative work due to myocardial lengthening during isovolumetric relaxation. Global wasted work (GWW) is the sum of myocardial lengthening in systole and shortening in isovolumetric relaxation, reflecting the work that does not contribute to LV ejection. Global work efficiency (GWE) is constructive work divided by the sum of constructive work and wasted work.

### Cardiac Troponin I (cTnI) measurements

The High sensitivity cTnI assay (STAT) from Abbott Diagnostics was used for the measurement of cTnI, before exercise and at 3 and 24 h following exercise. The assay was analyzed on an Architect SR2000i (Abbott Diagnostics, Abbott Park, IL, USA). Overall 99th percentile is 26 ng/L (men: 34 ng/L and women: 16 ng/L) (IFCC Committee on Clinical Applications of Cardiac Bio-Markers).

### Statistical analysis

Normally distributed continuous variables are reported as mean ± SD, while continuous variables with markedly skewed distributions are reported as the median and interquartile range (25th, 75th percentile). The Shapiro–Wilk test was used to test for normality. For continuous variables, The Mann–Whitney *U* test or a Student *T*-test was used to compare groups, as appropriate. Spearman analysis was used to calculate bivariate correlations. A two-tailed *p*-value < 0.05 was considered significant. SPSS version 26.0.0.1 was used for statistical analyses.

## Results

A total of 59 healthy, well-trained, recreational athletes, 52 (43, 59) years of age, 73% males, without obstructive coronary artery disease (> 50% stenosis) verified by CT scans, were included in the study (Table 1). The study participants had a median training volume of 61 (47, 102) MET hours/week and a median training experience of 10 (7, 21) years.

**TABLE 1:** Baseline characteristics (*n* = 59).

Characteristic	
Age, years	51.1 ± 9.7
Male sex, *n* (%)	43 (72.9)
BMI, kg/m^2^	24.9 (23.3, 27.1)
Waist circumference, cm	85.0 (81.0, 93.0)
SBP, mmHg	135 ± 16
DBP, mmHg	82 ± 10
Former smoker, *n* (%)	27 (44.3)
Family history of CVD	4 (6.6)
Prior CVD, *n* (%)	0
Diabetes, *n* (%)	0
**Training experience**	
Years of endurance training	10.0 (7.0, 21.3)
Number of prior competitions	10.0 (5.0, 20.0)
Exercise volume (MET hours/week)	61.3 (47.1, 101.7)
**Cardiopulmonary exercise test (CPET)**	
Max VO2 (mL/min/kg)	41.2 ± 8.4
Power at max VO2 (watt/kg)	3.8 ± 0.8
HR at VO2 max (bpm)	177 ± 12
Power at AT (watt)	200 ± 47
HR at AT (bpm)	162 ± 13

Normally distributed values are reported as mean ± SD, and markedly skewed values are reported as median (25th, 75th percentile). BMI, body mass index; SBP, systolic blood pressure; DBP, diastolic blood pressure; CVD, cardiovascular disease; MET, metabolic equivalent; HR, heart rate; AT, anaerobic threshold.

Before CPET (baseline), the mean SBP (systolic blood pressure) was 136 ± 16 mmHg, and the heart rate (HR) was 59 ± 10 bpm. Echocardiographic recordings at baseline showed a mean LV mass index of 87.5 ± 14.5 g/m^2^ and a mildly elevated median LV end-diastolic volume index of 82 (69, 97) mL/m^2^ compared to reference values (22). All other baseline echocardiographic measurements were within normal ranges.

### The impact of exercise on physiological and echocardiographic parameters

Both CPET and the race exercises were of high physical intensity (Table 2). There was a highly significant increase in cTnI after both exercises, with the highest values at 3 h after exercise, with declining values at 24 h (Table 2).

**TABLE 2:** Physical measurements cardiopulmonary exercise test (CPET) and race (*n* = 59).

Exercise parameters	CPET	Race	*P*-value
Duration of exercise (min)	43 (40, 45)	230 (210, 245)	<0.001
ATHR (bpm)	162 ± 13		
AT (Watt)	200 ± 47		
HR peak (bpm)	177 ± 12	175 ± 12	0.08
HR mean (bpm)	132 ± 12	154 ± 10	<0.001
SBP peak (mmHg)	201 (181, 216)	230 (210, 245)	<0.001
SBP mean (mmHg)	183 ± 14	166 ± 15	<0.001
DBP peak (mmHg)	83 (68, 94)	100 (90, 110)	<0.001
DBP mean (mmHg)	83 ± 9	84 ± 8	0.54
Power (watt/kg), peak	3.8 ± 0.8	9.2 ± 6.8	0.001
Power (watt/kg), mean	2.9 ± 0.5	2.1 ± 0.5	<0.001
Work total (watt × min)	8650 ± 1778	40289 ± 7714	<0.001
Work/kg total (watt/kg × min)	107 ± 21	496 ± 65	<0.001
Weight reduction (kg)	0.4 (0.2, 0.6)	1.3 (0.8, 1.8)	<0.001
Delta creatinine 3 h (μmol/L)	2.7 ± 4.5	11.0 ± 12.7	<0.001
**cTnI**			
Pre-exercise (ng/L)	3.2 (2.0, 7.0)	4.0 (2.1, 7.5)	0.77
3-h post-exercise (ng/L)	11.6 (7.0, 23.2)	77.1 (37.1, 128)	<0.001
24-h post-exercise (ng/L)	5.0 (2.9, 9.5)	15.7 (7.7, 32.1)	<0.001

Normally distributed values are reported as mean ± SD, and skewed values are reported as median (25th, 75th percentile). AT, anaerobic threshold; HR, heart rate; bpm, beats per minute; SBP, systolic blood pressure; DBP, diastolic blood pressure; cTnI, Cardiac Troponin I.

Immediately after exercise, SBP and DBP (diastolic blood pressure) decreased, but HR increased, and so did cardiac output (CO) and mechanical dispersion (Table 3). Most echocardiographic parameters were lower than pre-exercise values immediately following exercise, including LV stroke volume (SV), left atrial volume index (LAVi), LV diastolic and systolic volumes (EDV and ESV), E/A ratio, E, E^‵^, E/E^‵^, LV GLS, GWI, and GCW (Table 4). There were no significant differences in LVEF following exercise.

**TABLE 3:** Hemodynamic and echocardiographic parameters (*n* = 59).

		CPET	Race	*P*-value
**Blood pressure**				
SBP (mmHg)	Baseline	135 (122, 146)	143 (129, 156)	<0.001
	Post-exercise	128 (119, 136)	128 (122, 139)	0.037
	24 h	126 (119, 137)	135 (126, 151)	<0.001
DBP (mmHg)	Baseline	81 (74, 89)	78 (71, 87)	0.32
	Post-exercise	78 (70, 84)	72 (68, 81)	0.10
	24 h	78 (69, 86)	73 (69, 81)	0.08
Heart rate (bpm)	Baseline	58 (51, 65)	57 (50, 66)	0.78
	Post-exercise	79 (70, 90)	87 (83, 95)	<0.001
	24 h	56 (50, 63)	55 (50, 66)	0.74
**LV volume 3D**				
EDVi (mL/m^2^)	Baseline	81.6 (69.1, 96.5)	81.4 (70.3, 90.3)	0.32
	Post-exercise	79.8 (72.0, 88.9)	73.8 (64.2, 82.1)	<0.001
	24 h	92.1 (85.4, 103.3)	88.1 (80.3, 100.0)	<0.001
ESVi (mL/m^2^)	Baseline	33.8 (29.1, 39.4)	32.8 (28.0, 37.6)	0.018
	Post-exercise	34.2 (31.7, 39.8)	30.5 (26.3, 36.5)	<0.001
	24 h	39.4 (35.9, 42.9)	36.3 (32.9, 40.8)	0.002
**LV function**				
E/A ratio	Baseline	1.3 (1.1, 1.7)	1.2 (1.1, 1.6)	0.07
	Post-exercise	1.0 (0.9, 1.2)	0.9 (0.8, 1.1)	<0.001
	24 h	1.4 (1.1, 1.6)	1.3 (1.1, 1.7)	0.26
E^‵^ septal (m/sek)	Baseline	0.11 (0.09, 0.12)	0.12 (0.10, 0.14)	<0.001
	Post-exercise	0.10 (0.09, 0.12)	0.11 (0.08, 0.12)	0.29
	24 h	0.11 (0.09, 0.13)	0.11 (0.09, 0.14)	0.002
E/E^‵^ septal	Baseline	7.0 (6.0, 8.4)	6.9 (6.1, 7.9)	0.048
	Post-exercise	6.6 (5.5, 7.9)	6.6 (5.2, 8.3)	0.95
	24 h	7.2 (6.4, 8.9)	7.0 (6.0, 9.0)	0.38
EF (%)	Baseline	59.0 (55.0, 60.0)	58.0 (56.0, 63.0)	0.10
	Post-exercise	57.0 (55.0, 60.0)	58.0 (55.0, 61.5)	0.08
	24 h	58.0 (56.0, 60.0)	59.0 (56.0, 61.0)	0.28
SVi (mL/m^2^)	Baseline	43.0 (38.9, 52.0)	47.6 (42.8, 57.4)	<0.001
	Post-exercise	39.7 (34.4, 46.6)	42.8 (38.0, 50.1)	0.014
	24 h	43.3 (37.3, 50.6)	46.0 (41.6, 51.6)	0.001
CO (mL/min/m^2^)	Baseline	2610 (2151, 3055)	2907 (2446, 3268)	0.005
	Post-exercise	3328 (2724, 3831)	3772 (3311, 4379)	<0.001
	24 h	2388 (2178, 2814)	2575 (2260, 3053)	0.008
GLS (%)	Baseline	20.0 (18.0, 22.5)	20.8 (19.0, 22.4)	0.21
	Post-exercise	19.0 (17.0, 20.0)	19.5 (18.0, 22.0)	0.013
	24 h	20.0 (19.0, 22.0)	20.0 (18.0, 22.0)	0.54
Mechanical dispersion (msec)	Baseline	33.5 (29.0, 40.0)	31.5 (22.5, 40.8)	0.014
	Post-race	39.0 (28.0, 49.0)	36.0 (31.0, 43.0)	0.64
	24 h	35.0 (27.0, 41.5)	31.0 (24.5, 37.5)	0.025
**Left atrial volume 3D**				
EDVi (mL/m^2^)	Baseline	31.7 (25.0, 38.6)	30.6 (23.0, 37.8)	0.17
	Post-exercise	26.2 (22.9, 33.3)	23.7 (19.9, 27.5)	<0.001
	24 h	30.8 (25.1, 35.8)	31.8 (27.5, 39.0)	0.24
**RV**				
RV base diameter, mm	Baseline	40.0 (36.8, 42.0)	41.0 (37.8, 44.0)	0.12
	Post-exercise	39.0 (36.0, 42.0)	39.0 (35.5, 42.0)	0.43
	24 h	39.0 (37.0, 41.8)	40.0 (38.0, 42.0)	0.019
RV GLS, 3 segments, %	Baseline	27.0 (24.7, 28.8)	26.6 (23.8, 29.6)	0.98
	Post-exercise	25.9 (21.3, 28.6)	25.6 (22.6, 30.0)	0.19
	24-h	27.0 (25.1, 29.1)	25.9 (21.3, 28.6)	0.64

Values are median (25th, 75th percentile). SBP, systolic blood pressure; DBP, diastolic blood pressure; LV, left ventricle; EDVi, end-diastolic volume index; ESVi, end-systolic volume index; EF, ejection fraction; SVi, stroke volume index; CO, cardiac output; GLS, global longitudinal strain; RV, right ventricle.

**TABLE 4:** Myocardial work parameters (*n* = 59).

		CPET	Race	*P*-value
GWI (mmHg%)	Baseline	2156 (1899, 2399)	2493 (2192, 2638)	<0.001
	Post-exercise	1865 (1621, 2201)	2073 (1889, 2391)	<0.001
	24-h	2011 (1764, 2208)	2312 (2104, 2496)	<0.001
GCW (mmHg%)	Baseline	2383 (2152, 2668)	2601 (2360, 2811)	0.006
	Post-exercise	2096 (1972, 2434)	2252 (2036, 2580)	0.005
	24-h	2356 (2096, 2641)	2497 (2254, 2676)	0.43
GWW (mmHg%)	Baseline	66.0 (37.5, 128.0)	53.0 (36.0, 81.0)	0.06
	Post-exercise	58.0 (41.8, 104.3)	82.0 (41.5, 129.0)	0.21
	24-h	49.0 (33.5, 88.0)	49.0 (30.8, 66.8)	0.12
GWE (%)	Baseline	97.0 (95.0, 98.0)	98.0 (96.0, 98.0)	0.024
	Post-exercise	96.0 (95.0, 98.0)	96.0 (94.8, 98.0)	0.83
	24-h	97.0 (95.0, 98.0)	97.0 (97.0, 98.0)	0.012
PSS (%)	Baseline	−0.2 (−0.4, −0.1)	−0.4 (−0.9, 0.13)	0.009
	Post-exercise	−0.4 (−0.7, −0.2)	−0.6 (−2.1 to 0, 0.2)	0.005
	24-h	−0.1 (−0.3, −0.1)	−0.2 (−0.4, −0.1)	0.75
PSI (%)	Baseline	1.2 (0.6, 1.9)	2.0 (0.7, 5.0)	0.015
	Post-exercise	2.2 (1.1, 3.6)	2.7 (1.2, 11.5)	0.010
	24-h	0.7 (0.4, 1.6)	0.8 (0.4, 1.7)	0.98
ESL (%)	Baseline	1.7 (1.0, 2.8)	1.6 (0.9, 2.1)	0.28
	Post-exercise	2.7 (1.6, 3.8)	2.0 (1.2, 3.7)	0.07
	24-h	1.4 (0.8, 2.4)	1.8 (0.9, 3.0)	0.17

Values are median (25th, 75th percentile). GWI, global work index; GCW, global constructive work; GWW, global wasted work; GWE, global work efficiency; PSS, post systolic shortening; PSI, post systolic shortening index; ESL, early systolic lengthening.

Global wasted work (GWW) was significantly higher after the race (*p* 0.001) compared with the pre-exercise value and global work efficiency (GWE) was lower (*p* 0.006), this was not observed following the CPET. Twenty-four hours after the race GWW and GWE returned to baseline values.

Echocardiography at 24 h showed increased LV EDV and ESV, but a decreased CO compared with pre-exercise values.

### The impact of increased exercise intensity and duration, comparison of race, and CPET results

The race was of higher workload and longer duration than the CPET, resulting in higher post-exercise cTnI values (Table 2). Immediately following exercise, there was a significant reduction in SBP and DBP for both exercises, and still significantly lower than pre-exercise values at 24 h (Supplementary materials 2, 3). The reduction in SBP from before to after exercise was significantly larger for the race than the CPET (*p* < 0.05). Post-race HR was only higher than CPET immediately after the race returning to baseline values, equal to the CPET values, at 24 h following the race (Table 3). At all time-points following the race, there were smaller diastolic and systolic volumes compared with the CPET, but higher SV, larger cardiac output, and larger GWI compared with the CPET (Tables 3, 4). There was a lower E/A ratio and smaller LAVi immediately following the race that returned to baseline after 24 h with no difference compared with CPET. There was a significant reduction in LV GLS immediately after both the CPET and the race (*p* < 0.05), with a similar reduction after both exercises. LVEF did not change after the CPET nor after the race.

There were no significant correlations between echocardiographic parameters and cTnI after the CPET. There was a weak positive correlation between cTnI and end systolic- and diastolic volumes 24 h after the race (Spearman’s ρ 0.26 and 0.29, *p* = 0.05 and *p* = 0.03). There was also a positive correlation between LV GLS immediately after the race and cTnI 3 h after the race (Spearman’s ρ 0.275, *p* = 0.044) and 24 h after the Race (Spearman’s ρ 0.298, *p* = 0.028). There were no significant correlations between cTnI, LVEF, diastolic parameters, and MW parameters.

### Differences in pre-exercise measurements between the CPET and the race

Comparing the pre-exercise assessment before the CPET and the race revealed several significant differences (Tables 3, 4). Before the race, there was a significantly (*p* < 0.001) higher pre-exercise SBP than the CPET, but no difference in heart rate. A large portion of the echocardiographic findings before the race differed from pre-CPET findings (Tables 3, 4). The following echocardiographic values were higher before the race compared with the values before the CPET: CO (*p* = 0.005), LV SV (*p* < 0.001), E^‵^(*p* < 0.001), GWI (*p* < 0.001), GCW (*p* = 0.006), GWE (*p* = 0.024), and post systolic shortening index (*p* = 0.015). The following values were lower: ESV (*p* = 0.018), E/E^‵^ (*p* = 0.048), and post-systolic shortening (*p* = 0.009).

### Percentual changes from baseline to post-exercise

**FIGURE 2:**
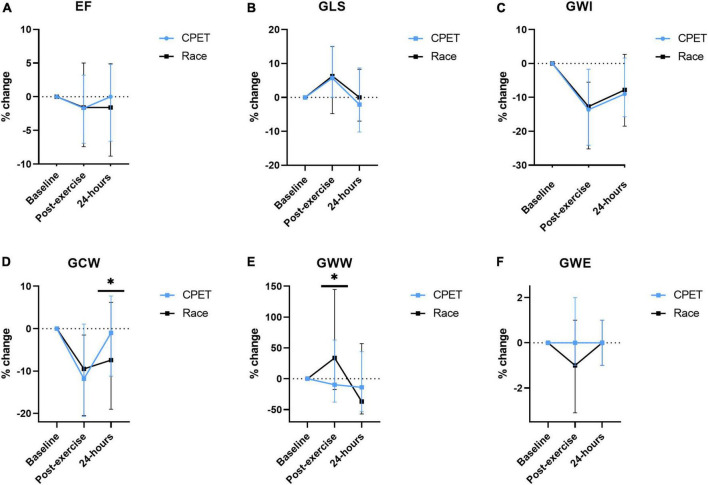
The percentual change of echocardiographic measurements from baseline (before exercise) and immediately and 24 h following a cardiopulmonary exercise test (CPET) and the North Sea Race in 2018 (Race). The following parameters were assessed **(A)** LVEF (left ventricular ejection fraction), **(B)** GLS (global longitudinal strain), **(C)** GWI (global work index), **(D)** GCW (global constructive work), **(E)** GWW (global wasted work), and **(F)** GWE (global work efficiency). *p < 0.05.

**FIGURE 3:**
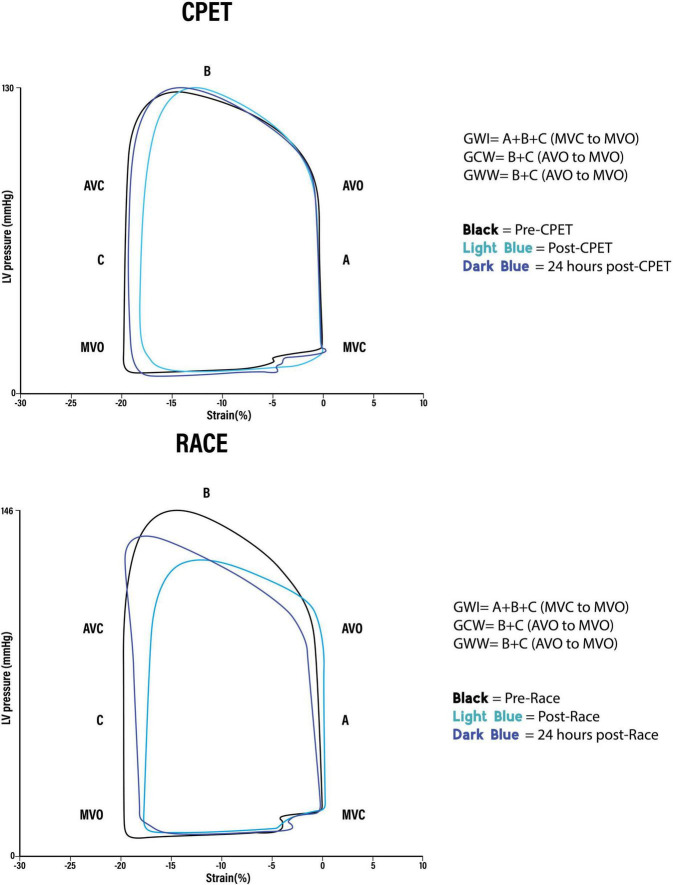
Representative global pressure-strain loops from the same person after (A) cardiopulmonary exercise test (CPET) and (B) Race. GWI, Global work index. Amount of myocardial work performed by the left ventricle during systole: Area of the pressure-strain loop from mitral valve closure to mitral valve opening (A + B + C). GCW, Global constructive work. Work that contributes to left ventricular ejection. Positive work performed in (B) systole (shortening) + negative work performed in (C) isovolumetric relaxation (lengthening). GWW, Global wasted work. Work that does not contribute to left ventricular ejection. Negative work performed in (B) systole (lengthening) + positive work performed in (C) isovolumetric relaxation (shortening).

The difference in many pre-exercise parameters before the CPET and the race indicates the presence of significant confounders complicating a direct comparison between the race and the CPET. In order to reduce the impact of baseline confounders, the difference in post-exercise parameters between the race and the CPET was assessed by the percentual change from each pre-exercise value (Figure 2). When using this approach, there were no significant post-exercise differences regarding LV EF, LV GLS, GWI, and GWE. However, there was increased GWW immediately after the race compared with the CPET (*p* = 0.025), and there was a significant persisting reduction in GCW 24 h following the race (*p* = 0.015). Figure 3 illustrates representative pressure-strain loops from one of the study subjects after the CPET and the race with a significant reduction in GWI immediately post-exercise for both CPET and the race, with a reduction in the pressure-strain loop area. Twenty-four hours after the CPET the area under the pressure strain loop is back to baseline area but still reduced 24 h after the race, illustrating a reduced ability to recover after the race.

### Reproducibility

Intra- and inter-observer variability analysis confirmed high reproducibility of the parameters LV GLS, four-chamber strain, three-chamber strain, and two-chamber strain analyses, and the myocardial work parameters GWI, GCW, GWW, and GWE (Supplementary material 4).

## Discussion

This is the first study to provide evidence suggesting myocardial inefficiency as an early indicator of exercise-induced myocardial fatigue. The study is based upon multiple echocardiographic assessments before and after exercise comparing two different workloads, in well-trained middle-aged athletes, without obstructive coronary artery disease verified by CT scans. The study found that increased exercise workload was associated with increased post-exercise wasted myocardial work without alterations in LVEF and GLS adjusting for baseline values. These findings suggest that markers of myocardial inefficiency may precede reduction in global LV function as markers of myocardial fatigue.

Exercise-induced cardiac fatigue is a reversible myocardial dysfunction caused by strenuous exercise (24, 25). A large range of parameters have been used to assess exercise-induced cardiac fatigue, the most common are LVEF and LV-GLS (8, 26, 27). Since echocardiographic parameters, such as LVEF and GLS are influenced by pre- and afterload, it is unknown whether post-exercise cardiac dysfunction represents a global vascular load-related mechanism or a more local intrinsic mechanism in the cardiomyocyte. The present study found exercise-induced changes in left ventricular function consistent with cardiac fatigue (8). LV GLS and GWI were reduced after both exercises. In contrast, there was a significant increase in wasted myocardial work (GWW) and mechanical dispersion after the highest workload (the race) compared with the CPET. The level of constructive myocardial work was reduced after both exercises and remained reduced at 24 h after the race but not after the CPET, suggesting that the increased workload during the race resulted in reduced LV recovery.

Myocardial work by echocardiography combines strain analysis with SBP providing a less load-dependent and more comprehensive assessment of LV systolic function (28). Myocardial work parameters may be used to evaluate athletes’ hearts (29) and to distinguish pathology from athletic remodeling (28, 30). A recent study of marathon runners found a post-exercise increase in myocardial work (GWI) in a subgroup of athletes with higher BNP values and heart rates (31). However, no study has determined the role of myocardial inefficiency in exercise-induced myocardial fatigue.

Muscular inefficiency occurs in skeletal muscle when workloads exceed maximal oxygen consumption intensity (32). The prolonged duration of these high-intensity workloads is linked with a progressive reduction in muscular efficiency (33). Exercise-induced skeletal muscular inefficiency is associated with electromechanical delay, potentially leading to exercise intolerance (34, 35). Several potential mechanisms are suggested to be related to muscular inefficiency, including muscle metabolite accumulation, decreased free energy of adenosine triphosphate breakdown, increased muscle temperature, and reactive oxygen species production (32).

Our echocardiographic findings suggest that there may be a similar relation between workload and myocardial inefficiency, as previously described in skeletal muscle. In our study, the increased workload and the duration of work during the race (Table 2) were associated with a post-exercise reduction in myocardial efficiency, indicated by parameters such as global wasted work (GWW), global constructive work (GCW), post-systolic shortening, and mechanical dispersion. These parameters of myocardial inefficiency may add additional information to traditional parameters of exercise-induced myocardial fatigue. The underlying mechanisms responsible for the observed exercise-induced cardiac inefficiency are unknown. However, exercise-induced alterations in cardiac electromechanical delay may represent similar mechanisms to those described in skeletal muscle. Chan-Dewar et al. (36) reported an increase in post-exercise electromechanical delay without changes in QRS duration, suggesting an intrinsic myocyte mechanism responsible for exercise-induced cardiac fatigue. Sahlèn et al. (37) found exercise-induced cardiac fatigue associated with abnormalities in ventricular repolarization, suggesting a transient state of electrical instability following endurance exercise.

Our study also found increases in other parameters of myocardial inefficiencies, such as post-systolic shortening (PSS) and early systolic lengthening (ESL) immediately after both exercises. Post-systolic shortening relates directly to LV systolic function (38). Myocardial contraction after aortic valve closure and lengthening of myocardial fibers during systole reflect paradoxical deformation of the myocardial wall segments, attenuating LV ejection. Since GWW considers SBP, it is less load-dependent than PSS and ESL and, therefore, a more accurate measure of LV efficiency. Post-race increase in GWW and the persistent reduction in GCW following the race may indicate an exercise-induced reduction in myocardial efficiency caused by the prolonged myocardial work during the race. In contrast, neither LV GLS nor LVEF showed any difference between the two types of exercise, despite a large increase in cTnI following the race, indicating increased myocardial stress (21). Interestingly, in line with recent publications (25), there was a significant increase in mechanical dispersion following the race suggesting increased LV mechanical discoordination and reduced mechanical efficiency during cardiac ejection after prolonged high-intensity workload (39).

Left ventricular (LV) rotation and twist are important factors of LV systolic and diastolic performance. During diastole, the untwisting results in the abrupt release of the energy stored in elastic components, resulting in a negative intraventricular pressure gradient that facilitates LV filling at low filling pressure (40, 41). Park et al. (42) found that rotation and twist both showed higher values in the abnormal relaxation group than in the healthy group, paralleled by the reduced peak velocity of the early diastolic filling wave (E) and peak early diastolic annular velocity (E^‵^), similar to the findings in the present study. At the end of systole and during isovolumic relaxation, myocytes are still active and exert the force to oppose the ventricular pressure and residual elastic forces due to the twist, which is the highest at the apex. One may speculate that the faster the untwisting, the more negative intraventricular pressure is generated, thus unloading the myocytes, which can eventually result in the myocyte shortening during isovolumic relaxation and increased global wasted work (GWW). However, the exact mechanisms and consequences for the observed reduced cardiac inefficiency are unknown, and further work regarding the mechanisms underpinning the reduced efficiency following intense exercise is required.

In line with previous exercise studies, there were significant post-exercise reductions in 3D-derived LV end-diastolic, left atrial volumes, and alterations in diastolic function (6, 8, 43). A significantly increased post-exercise heart rate, reduced LV filling time and left-sided volumes may explain some of the observed alterations of the echocardiographic assessments performed just after exercise. The interactions between the left and right atria and ventricles are central in the post-exercise alterations in cardiac function (44). Altered loading conditions cause alterations in RV volumes and function with a subsequent impact on left atrial preload, LV filling, and LV function (43). In contrast to La Gerche et al. (43), the present study did not find significant alterations in RV function. There may be several explanations for this discrepancy. The study by La Gerche et al. found a more significant reduction in RV functions in athletes competing for a longer duration (up to 11 h). In the present study, exercise duration was significantly shorter. We, therefore, cannot exclude that alterations in right heart volumes and functions had an impact on the present findings.

The present study used two different workloads to assess the post-exercise response. The CPET was used to define the reference LV response to standardized exercise intensity, ensuring that all study individuals were exposed to an exercise of similar duration with the same metabolic demands (lactate threshold and max V02). Furthermore, the CPET allowed interpretation of the implications of the work performed during the race using power meters. The power meters indicated a substantial increase in the exercise above max VO2 level during the race, increasing the likelihood of developing myocardial inefficiency.

### Impact of pre-exercise stress on myocardial work parameters

As indicated by the significant increase in blood pressure the day before the race, the race and the preparation for the next day may have induced a stress response in the race contenders. To adjust for this increased stress, echocardiographic parameters were corrected by using the pre-exercise measurements as a baseline, and the percentual change from each pre-exercise value assessed the difference in post-exercise parameters.

The present study demonstrates pre-exercise stress’s impact on assessing resting echocardiographic parameters. SBP was higher before the race compared with the CPET. This increase in SBP affects the MW parameters, causing increased GWI and GCW compared with the assessment before the CPET. The increased blood pressure is most likely due to the mental stress caused by the competitive event, resulting in increased myocardial work before the race. The present study’s findings underscore the impact of emotional stress on myocardial work and the need to consider the influence of emotional stress when baseline MW parameters are interpreted.

### Limitations

The study provides insights into the longitudinal cardiac morphology changes and measures after prolonged exercise in middle-aged recreational athletes. The present study addresses morphological and functional changes in a population different from younger athletes, and the current findings may not apply to a younger population with a higher functional capacity. When calculating MW, we used SBP as a substitute for force; therefore, the use of pressure and strain does not provide a direct measure of work (wall thickness and radius of curvature are not included). Calculation of work is underestimated in dilated ventricles because of higher wall stress at any given LV pressure, and this could also be the case in our study, where study subjects had mildly dilated ventricles. Calculating the myocardial work parameters relies on global longitudinal strain measurements, systolic blood pressure, and valvular events. Small changes in the timing of valvular events could potentially lead to significant differences in the work parameters, especially the global wasted work and global work efficiency parameters. When assessing and evaluating these parameters, it is essential to be aware of these challenges.

## Conclusion

Changes in left ventricular function, consistent with exercise-induced fatigue, were seen after both exercises with the varying workload. However, increased endurance exercise duration and workload, as performed at the race, were associated with decreased myocardial efficiency following exercise. When correcting for alterations in baseline parameters, there was no difference in LVEF and GLS comparing the two exercise workloads and durations. These findings suggest that myocardial work parameters may be more sensitive measures of myocardial inefficiency than traditional markers of systolic myocardial dysfunction.

## Data availability statement

The raw data supporting the conclusions of this article will be made available by the authors, without undue reservation.

## Ethics statement

The studies involving human participants were reviewed and approved by the Regional Ethics Committee (REK nr 2013/550). The patients/participants provided their written informed consent to participate in this study. Written informed consent was obtained from the individual(s) for the publication of any potentially identifiable images or data included in this article.

## Author contributions

CE and SØ drafted the manuscript. CE, ØK, MB-B, VF, MC, LK, LD, TM, ØS, and SØ contributed to the data collection. CE and BA performed the statistical analyses. All authors contributed to drafting and critically revising the manuscript and approved the submitted version.
