# Supplementary material for: Myocardial inefficiency is an early indicator of exercise-induced myocardial fatigue
Source: Front Cardiovasc Med. 2023 Jan 11;9:1081664. doi: 10.3389/fcvm.2022.1081664 (PMC9874326; doi:10.3389/fcvm.2022.1081664)
Supplement: Supplementary file 1 [file Presentation_1.PDF]

**Supplement 1**

**Cardiopulmonary exercise test (CPET):**

All study participants were tested on their personal bikes fitted to a Cyclus 2 electronically braked ergotrainer (RBM elektronik-automation; Leipzig, GER). Each participant performed a 10-minute warm-up before exercise tests, resistance was kept low and was guided by the test-leader. The lactate threshold test was executed as a 4-minute incremental load stepwise test. The workload was based on previous training history and results from warm-up (min 50w – maximum 220w). The workload was increased with fixed individualized (min 15w – maximum 30w) steps every fourth minute. Lactate was measured in capillary blood from the participants' index finger on the Lactate Scout+ (EKF Diagnostic, Cardiff, GB). Gas exchange was measured breath by breath on a Jaeger Vyntus CPX (Carefusion, Hoechberg, GE). Lactate threshold was defined as a lactate value > 1.5 mmol/l above mean value from step 1 and 2 or a RER > 1.0. For each step, including rest and warm-up, the following variables were collected; Work (watt), blood pressure (mmHg), VO2 (ml/min/kg), RER, Lactate, and heart rate (bpm). Following the stepwise determination of lactate threshold, participants were allowed a maximum of 5-minute cooldown, before performing the VO2max test. The VO2max test was a ramp protocol started at 70-250 (min-max) watts with an increase in the workload of 15-32 (min-max) Watt/min until exhaustion. The VO2max test was performed to reach maximum effort between 5 and 10 minutes. Pre-test blood pressure was obtained at the start of the test and maximal blood pressure was obtained immediately after the end of the test with the participant still seated on the bike. VO2max was defined as the point where VO2 reached a plateau despite increasing resistance. Peak power and peak heart rate were the maximum value achieved during this test.
