# Supplementary material for: Myocardial inefficiency is an early indicator of exercise-induced myocardial fatigue
Source: Front Cardiovasc Med. 2023 Jan 11;9:1081664. doi: 10.3389/fcvm.2022.1081664 (PMC9874326; doi:10.3389/fcvm.2022.1081664)
Supplement: Supplementary file 2 [file Table_1.DOCX]

**Supplementary 2
Hemodynamic and echocardiographic parameters (n= 59)**

|  |  | **Pre exercise (n=59)** | **Post-exercise(n=59)** | **24 h post exercise (n=59)** | **P: Pre-post** | **P: Post -24 h** | **P: Pre-24 h** |
| --- | --- | --- | --- | --- | --- | --- | --- |
| **Physiological parameters** |  |  |  |  |  |  |  |
| SBP, mmHg | Race | 143.0 (129.0,156.0) | 128.0 (122.0,139.0 | 135.0 (126.0,151.0) | <0.001 | <0.001 | 0.001 |
|  | CPET | 135.0 (122.0-146.0) | 128.0 (119.0,136.0) | 126.0 (119.0,137.0) | <0.001 | 0.81 | <0.001 |
| DBT, mmHg | Race | 78.0 (71.0,87.0) | 72.0 (68.0,81.0) | 73.0 (69.0,81.0) | 0.002 | 0.75 | 0.002 |
|  | CPET | 81.0 (74.0,89.0) | 78.0 (70.0,84.0) | 78.0 (69.0,86.0) | <0.001 | 0.88 | <0.001 |
| Heart rate, bpm | Race | 57.0 (50.0,66.0) | 87.0 (83.0,95.0) | 55.0 (50.5,65.5) | <0.001 | <0.001 | 0.108 |
|  | CPET | 58.0 (51.5,65.5) | 79.0 (70.0,90.0) | 56.0 (50.5,63.0) | <0.001 | <0.001 | 0.108 |
|  |  |  |  |  |  |  |  |
| **LV internal dimensions (mm)** |  |  |  |  |  |  |  |
| Diastole | Race | 49.0 (45.0,53.0) | 48.0 (44.0,51.0) | 47.0 (45.0,52.0) | 0.001 | 0.252 | 0.045 |
|  | CPET | 48.0 (45.0,52.0) | 46.5 (44.0,51.0) | 47.0 (45.0,52.0) | 0.01 | 0.52 | 0.17 |
| Systole | Race | 32.0 (29.0,35.0) | 30.0 (28.0,34.0) | 30.0 (28.0,34.3) | 0.008 | 0.241 | 0.021 |
|  | CPET | 31.0 (29.0,32.3) | 33.0 (29.0,35.0) | 33.0 (30.0,36.0) | 0.74 | 0.297 | 0.191 |
| **LV volume (ml/m2) 3D** |  |  |  |  |  |  |  |
| Diastole | Race | 81.4 (70.3,90.3) | 73.8 (64.2,82.1) | 88.1 (80.3,100.0) | 0.002 | <0.001 | 0.001 |
|  | CPET | 81.6 (69.1,96.5) | 79.8 (72.0,88.9) | 92.1 (85.4,103.3) | 0.27 | <0.001 | <0.001 |
| Systole | Race | 32.8 (28.0,37.6) | 30.5 (26.3,36.5) | 36.3 (32.9,40.8) | 0.09 | <0.001 | <0.001 |
|  | CPET | 33.8 (29.1,39.4) | 34.2 (31.7,39.8) | 39.4 (35.9,42.9) | 0.07 | <0.001 | <0.001 |
| **LV Function** |  |  |  |  |  |  |  |
| E/A ratio | Race | 1.2 (1.1,1.6) | 0.9 (0.8,1.1) | 1.3 (1.1,1.7) | <0.001 | <0.001 | 0.038 |
|  | CPET | 1.3 (1.1,1.7) | 1.0 (0.9,1.2) | 1.4 (1.1,1.6) | <0.001 | <0.001 | 0.443 |
| E` septalt, m/sek | Race | 0.12 (0.10,0.14) | 0.11 (0.08,0.12) | 0.11 (0.09,0.14) | <0.001 | 0.022 | 0.22 |
|  | CPET | 0.11 (0.09,0.12) | 0.10 (0.09,0.12) | 0.11 (0.09,0.13) | 0.019 | 0.226 | 0.31 |
| E/E` septalt | Race | 6.9 (6.1,7.9) | 6.6 (5.2,8.3) | 7.0 (6.0,9.0) | 0.17 | 0.112 | 0.172 |
|  | CPET | 7.0 (6.0,8.4) | 6.6 (5.5,7.9) | 7.2 (6.4,8.9) | 0.002 | 0.001 | 0.55 |
| LV Ejection fraction, 3D, ( %) | Race | 58.0 (56.0,63.0) | 58.0 (55.0,61.5) | 59.0 (56.0,61.0) | 0.40 | 0.80 | 0.378 |
|  | CPET | 59.0 (55.0,60.0) | 57.0 (55.0,60.0) | 58.0 (56.0,60.0) | 0.20 | 0.27 | 0.814 |
| Stroke volume index (ml/m2) | Race | 47.6 (42.8,57.4) | 42.8 (38.0,50.1) | 46.0 (41.6,51.6) | <0.001 | 0.07 | 0.006 |
|  | CPET | 43.0 (38.9,52.0) | 39.7 (34.4,46.6) | 43.3 (37.3,50.6) | <0.001 | 0.025 | 0.199 |
| LVCO (ml/min/m2) | Race | 2907 (2446,3268) | 3772 (3311,4379) | 2575 (2260,3053) | <0.001 | <0.001 | 0.006 |
|  | CPET | 2610 (2151,3055) | 3328 (2724,3831) | 2388 (2178,2814) | <0.001 | <0.001 | 0.019 |
| LV GLS (%) | Race | 20.8 (19.0,22.4) | 19.5 (18.0,22-0) | 20.0 (18.0,22.0) | 0.015 | 0.123 | 0.161 |
|  | CPET | 20.0 (18.0,22.5) | 19.0 (17.0,20.0) | 20.0 (19.0,22.0) | <0.001 | <0.001 | 0.024 |
| Mechanical dispersion ( msec) | Race | 31.5 (22.5,40.8) | 36.0 (31.0,43.0) | 31.0 (24.5,37.5) | 0.009 | 0.005 | 0.324 |
|  | CPET | 33.5 (29.0,40.0) | 39.0 (28.0,49.0) | 35.0 (27.0,41.5) | 0.29 | 0.23 | 0.993 |
| Left atrium |  |  |  |  |  |  |  |
| Volume, ml/m2 | Race | 30.6 (23.0,37.8) | 23.7 (19.9,27.5) | 31.8 (27.5,39.0) | <0.001 | <0.001 | <0.001 |
|  | CPET | 31.7 (25.0,38.6) | 26.2 (22.9,33.3) | 30.8 (25.1,35.8) | <0.001 | <0.001 | 0.65 |

Values are median (25^th^,75^th^ percentile). SBP, systolic blood pressure; DBP, diastolic blood pressure; LV, left ventricle; EF, ejection fraction; CO, Cardiac output; GLS: Global Longitudinal Strain.
