# Supplementary material for: Myocardial inefficiency is an early indicator of exercise-induced myocardial fatigue
Source: Front Cardiovasc Med. 2023 Jan 11;9:1081664. doi: 10.3389/fcvm.2022.1081664 (PMC9874326; doi:10.3389/fcvm.2022.1081664)
Supplement: Supplementary file 3 [file Table_2.DOCX]

**Supplemetary 3, Myocardial work parameters (n= 59)**

|  | **Type of Exercise** | **Pre exercise** | **Post exercise** | **24h post exercise** | **P-value** | **P- value** | **P-value** |
| --- | --- | --- | --- | --- | --- | --- | --- |
|  |  |  |  |  | **Pre-post** | **post-24h** | **pre-24h** |
| **GWI** | **CPET** | 2156.0 (1899.0,2399.5) | 1865.0 (1621.5,2201.0) | 2011.0 (1764.0,2208.5) | <0.001 | 0.04 | 0.003 |
| **GWI** | **Race** | 2493.0 (2192.0, 2638.0) | 2073.0 (1888.5, 2390.5) | 2312.0 (2103.5, 2496.0) | <0.001 | 0.044 | 0.001 |
| **GCW** | **CPET** | 2383.0 (2151.5,2668.0) | 2096.0 (1972.3,2434.5) | 2356.0 (2095.5,2641.5) | <0.001 | <0.001 | 0.48 |
| **GCW** | **Race** | 2601.0 (2360,2811.0) | 2252.0 (2035.8,2580.5) | 2497.5 (2254.0,2676.5) | <0.001 | 0.042 | 0.003 |
| **GWW** | **CPET** | 66.0 (37.5,128.0) | 58.0 (41.8,104.3) | 49.0 (33.5,88.0) | 0.68 | 0.91 | 0.46 |
| **GWW** | **Race** | 53.0 (36.0,81.0) | 82.0 (41.5,129.0) | 49.0 (30.8,66.8) | 0.001 | 0.004 | 0.92 |
| **GWE** | **CPET** | 97.0 (95.0,98.0) | 96.0 (95.0,98.0) | 97.0 (95.0,98.0) | 0.50 | 0.42 | 0.66 |
| **GWE** | **Race** | 98.0(96.0,98.0) | 96.0 (94.8,98.0) | 97.0 (97.0,98.0) | 0.006 | 0.002 | 0.34 |

Values are median (25^th^,75^th^ percentile). GWI, global work index; GCW, global constructive work; GWW, global wasted work; GWE, global work efficiency.
