# Supplementary material for: Myocardial inefficiency is an early indicator of exercise-induced myocardial fatigue
Source: Front Cardiovasc Med. 2023 Jan 11;9:1081664. doi: 10.3389/fcvm.2022.1081664 (PMC9874326; doi:10.3389/fcvm.2022.1081664)
Supplement: Supplementary file 4 [file Table_3.DOCX]

**Supplemetary 4**

**Reproducibility**

|  | **Intraobserver reproducibility** | | **Interobserver reproducibility** | |
| --- | --- | --- | --- | --- |
|  | ICC (95% CI) | CV | ICC (95% CI) | CV |
| GLS average | 0.954 (0.879-0.978) | 2.6% | 0.850 (0.433-0.962) | 1.5% |
| GLS A4 C | 0.951 (0.839-0.986) | 2.5% | 0.780 (0.204-0.944) | 2.6% |
| GLS A2C | 0.988 (0.969-0.994) | 1.5% | 0.890 (0.550-0.973) | 3.3% |
| GLSA3C | 0.964 (0.839-0.986) | 2.6% | 0.906 (0.609-0.977) | 2.5% |
| GWI | 0.990 (0.962-0.997) | 1.3% | 0.988 (0.939-0.998) | 2.0% |
| GCW | 0.985 (0.942-0.996) | 1.9% | 0.993 (0.965-0.999) | 1.9% |
| GWW | 0.998 (0.992-0.999) | 2.9% | 0.972 (0.860-0.994) | 7.4% |
| GWE | 0.920 (0.701-0.978) | 2.7% | 0.830 (0.149-0.966) | 2.7% |

GLS, global longitudinal strain; A4C, apical four-chamber; A2C, apical two chamber; A3C, apical three chamber; GWI, global work index; GCW, global constructive work; GWW, global wasted work; GWE, global work efficiency; ICC, intraclass correlation coefficient; CV, coefficicent of variation.
